# Neutrophils alter the metabolic profile of a coculture model of triple-negative breast cancer and stromal cells

**DOI:** 10.1186/s12885-026-17186-6

**Published:** 2026-10-22

**Authors:** Anna Thunström Salzer, Chloe Williams, Samuel Skoluda, Marios Stylianou, Jonathan D. Gilthorpe, Constantin F. Urban

**Affiliations:** 1 Department of Clinical Microbiology, Umeå University, Umeå, Sweden; 2 Department of Diagnostics and Intervention, Umeå University, Umeå, Sweden; 3 Department of Medical and Translational Biology, Umeå University, Umeå, Sweden; 4 Umeå Centre for Microbial Research (UCMR), Umeå University, Umeå, Sweden

**Keywords:** Triple-negative breast cancer, Neutrophils, Coculture, Mitochodrial activity, Inflammation

## Abstract

**Background:**

Breast cancer is one of the most common cancer types. Triple-negative breast cancer (TNBC), one of the most lethal breast cancer subtypes, is characterized by substantial neutrophil accumulation in the tumor microenvironment (TME), although the impact of neutrophils on disease progression remains unclear.

**Methods:**

To model interactions between cancer cells and stromal cells, we used a coculture system combining a TNB Ccell line with breast fibroblasts and exposed these cultures to neutrophils isolated from healthy donors.

**Results:**

Coculture generated a pro-inflammatory environment, with increased levels of pro-inflammatory cytokines resembling cytokine profiles reported in cancer patients. Coculture-conditioned medium attracted neutrophils and activated them to produce reactive oxygen species (ROS). Coculture also altered metabolic activity, as measured by the Seahorse Extracellular Flux Assay, compared with the corresponding monocultures.

**Conclusions:**

Adding neutrophils to the coculture increased mitochondrial activity, suggesting that neutrophils may promote a metabolic shift in the coculture system: Together, our results support further investigation of neutrophil-driven changes in energy metabolism in TNBC microenvironments.

**Supplementary information:**

The online version contains supplementary material available at https://doi.org/10.1186/s12885-026-17186-6.

## Background

Neutrophils are part of the innate immune system and are the most abundant circulating leukocytes [1]. Neutrophils migrate to sites of infection along chemokine gradients [2] where they eliminate microbes [3]. As part of the activation process, neutrophils produce reactive oxygen species (ROS), which damage pathogens and serve as signaling molecules [4]. In addition, neutrophils release web-like structures termed neutrophil extracellular traps (NETs), which are composed of chromatin fibers decorated with granular and cytosolic proteins, such as neutrophil elastase, myeloperoxidase, and S100A8/A9 [5, 6]. Neutrophil phenotypes show considerable plasticity, allowing these cells to adapt to different conditions, including inflammation, infection, cancer, and hypoxia [7]. Cancer patients often have elevated circulating neutrophil levels [8], but the contribution of neutrophils to cancer immunity and cancer progression appears to depend on cancer origin and tissue context [9, 10]. In some settings, neutrophils display anti-tumorigenic phenotypes that promote tumor killing [7], whereas in other settings they acquire tumor-promoting and immunosuppressive functions associated with disease progression, as reported for lung adenocarcinoma [11], or even worse overall survival [12]. A subgroup of neutrophils, namely low-density neutrophils (LDNs), are active during cancer and have immunosuppressive properties [13].

Triple-negative breast cancer (TNBC) is defined by the absence of estrogen receptor and progesterone receptor expression and lack of human epidermal growth factor receptor 2 (HER2) overexpression [14]. The absence of these therapeutic targets limits treatment options and contributes to the aggressive clinical manifestation of TNBC [15]. Neutrophils are more frequently present in the tumor microenvironment of TNBC than in other breast cancer subtypes [16]. Neutrophil accumulation during TNBC very likely affects the TME, by the release of cytokines and ROS, for instance, which in turn will change the metabolic state of present cancer and tissue cells, yet the functional consequences of this accumulation remain largely unknown.

To study alterations of cellular metabolism during cancer coculture systems with cancer and stromal cells have been established [17]. In the 1920s, Otto Warburg proposed that tumor cells rely heavily on glycolysis as an energy source, a concept later termed the Warburg effect [18]. Tumors consist of multiple cell types, among which stromal fibroblasts are common components [19]. Less than two decades ago, the reverse Warburg effect was proposed [20]. This concept suggests that glycolytic processes in the TME may be driven primarily by stromal cells, including fibroblasts, rather than by tumor cells themselves. Communication of cancer cells and surrounding fibroblasts can alter metabolic interactions for the benefit of cancer cells and at the expense of fibroblasts [21]. Oxidative stress in cancer cells appears to induce the metabolic reprogramming of fibroblasts [21].

Here, we used a coculture system combining a TNBC cell line with fibroblasts from healthy breast tissue, in the presence or absence of neutrophils, to examine neutrophil responses in this setting. For this purpose, healthy donorneutrophils were added to monocultures and cocultures to investigate their effect on cytokine landscapes, neutrophil activation, metabolic circuits, and tumor cell growth.

## Methods

### Cell culture

We used a fibroblast cell line derived from healthy breast tissue and MDA-MB-231, a cell line (Table 1). MDA-MB-231 cells have been extensively characterized as a basal-like cell line [22]. Both, cancer and fibroblast cell lines were kindly provided by Professor Michael P. Lisanti, University of Salford, Manchester. Authenticity of fibroblast and MDA-MB-231 cells was verified by morphological features, expression of the correct fluorescent markers, the cytokine profile during the coupling effect in cocultures, and STR analysis (CD Genomics) resulting in authentic STR profiles. Cell lines were frozen in aliquots upon arrival and fresh cultures were prepared from frozen stock when 3 passages were surpassed. All cell lines were intermittently tested (in-house) for Mycoplasma contamination for the duration of the project using 16s rRNA-specific primers. Cells were cultured in T75 cell culture flasks with 20 ml of Dulbecco’s modified Eagle medium (DMEM; #12491015 Thermo Fisher Scientific) supplemented with 100 units/ml penicillin, 100 µg/ml streptomycin, and 10% heat-inactivated filtered fetal bovine serum (#A5670701 Thermo Fisher Scientific) in a humidified incubator at 37 °C and 5% CO_2_. When cells reached confluence, they were passaged using 0.05% trypsin/EDTA. Viable cells were counted using a Bio-Rad TC20 automated cell counter with trypan blue. Cocultures were established in 12-well plates at a 2:1 ratio of fibroblasts to cancer cells, similarly as described previously [23]. More specifically we used 6.6 × 10^4^ fibroblasts and 3.3 × 10^4^ cancer cells in a total volume of 800 µl DMEM supplemented with 10% Nu-serum growth medium supplement (#CB 51000 Fisher Scientific) per well. Wells containing only fibroblasts or only cancer cells were prepared as controls. After 72 h, 1 × 10^5^ neutrophils were added to each well in a volume of 200 µl. After 6 h of incubation, conditioned media were collected and centrifuged for 5 min at 300 × g at room temperature to pellet remaining neutrophils. The resulting conditioned media were stored at -80 °C until cytokine analyses were performed. Throughout the study, we used a fibroblast to MDA-MB-231 to neutrophil ratio equaling 2:1:3 as determined by pilot experiments. These were guided by observations of neutrophil-mediated effects on cytokine profiles and metabolic assays without having adverse effects, such as cell detachment (data not shown).

**Table 1:** Details about the fibroblast cell line and the TNBC cell line used in the study including original publications. Ref. = Literature reference.*

Cell type	Disease	Origin	Organ	Source	Cell line name	Genetic modification	Ref.
Fibroblast	Healthy tissue	Connective tissue	Mammary tissue	Mammary tissue	218	Immortalized with hTERT, GFP-expression and puromycin resistance (retroviral construct)	[24]
Epithelia	Adeno-carcinoma	Breast	Mammary gland	Pleural effusion	MDA-MB-231cherry (DSMZ; ACC-982)	Expressing mCherry, transduction performed with a lentiviral vector*	[22, 25]

### Neutrophil isolation

Blood was collected in EDTA-containing tubes from healthy blood donors during routine blood donation at the Blood Centre. Written and/or oral consent was obtained by Blood Centre staff according to the principles of the Declaration of Helsinki. Donations were anonymous to the person performing neutrophil isolation, and no donor information was collected. Neutrophils were isolated using Histopaque-119 (#11191 Sigma-Aldrich) separation followed by a Percoll (#17089101 Cytiva) gradient, according to a previously described procedure [26]. This isolation protocol usually generates > 90% mature neutrophils [27, 28]. After isolation, viable cells were counted using a Bio-Rad TC20 automated cell counter with trypan blue.

### Cytokine analysis

Samples of conditioned media were thawed on ice. A Luminex ELISA assay with a magnetic bead washing system (Bio-Plex Pro-assay #12007283, BioRad) was used and the assay was carried out according to the manufacturer´s protocol. Conditioned media from 4 biological replicates were run with one technical replicate per sample.

### Chemotaxis assays

Neutrophils were resuspended in RPMI (#50-220-9736 RPMI 1640, Lonza) with 0.05% human serum albumin (HSA, Alburex© 5, CSL Behring) and stained with 3.3 µM bis-2-carboxyethyl-5-[and-6]-carboxyfluorescein-AM (#C3411 Sigma-Aldrich) at room temperature for 20 min. The cells were then washed and resuspended in RPMI with 0.05% HSA. Cells (5 × 10^5^) were seeded in a fluorescence impermeable transwell insert system (BD Falcon, HTS FluoroBlok #35112, 3 μm pore size, PET membrane). The transwells were placed in a 24-well plate with wells loaded with 300 µl of conditioned media collected from coculture or cells cultured alone (as described previously) mixed with 300 µl of DMEM. DMEM with 10 nM formal-methionyl-leucyl-phenylalanine (fMLF, #47729 Sigma-Aldrich) was added as positive control and DMEM alone as an unstimulated control. One technical replicate for each condition except for fMLF, which was run with two technical replicates. Neutrophils seeded directly into the well served as maximum level control (set as 100%). Total migration was determined by measuring the fluorescence intensity at 30 min, expressed as a percentage of the maximum level, after subtracting the baseline intensity of the media alone.

### ROS production

We used a luminescence-based assay to measure ROS production. Neutrophils (5 × 10^4^) were seeded in a white 96-well plate with 50 µM luminol (#123072, Sigma-Aldrich) and 1.2 U ml^− 1^ horseradish peroxidase (#SRE0082, Sigma-Aldrich). The plate was incubated for 15 min at 37 °C and 5% CO_2_ before conditioned media collected from fibroblast monocultures, cancer cell monocultures, or cocultures were added. DMEM with 10% Nu-serum (Fisher Scientific) was used as an unstimulated control. Neutrophils exposed to 20 nM phorbol 12-myristate 13-acetate (PMA, #79346 Sigma-Aldrich) were used as a positive control. Chemiluminescence was measured every second minute for 3 h at 37 °C using a microplate reader (Varioskan Flash, Thermo Fisher Scientific). Samples were run with 5 technical replicates for each condition and each value represents the mean of the five technical replicates. ROS production is presented as area under the curve (AUC) during the 3-hour measurement.

### Seahorse extracellular flux assays

A 96-well Seahorse cell culture plate was coated with 50 µg ml^− 1^ poly-D-lysine (#P7280 Sigma-Aldrich) and incubated for 30 min at room temperature 72 h prior to running the XFe96 assays. Coated plates were washed with PBS prior to seeding of cells; 8 × 10^3^ fibroblasts and 4 × 10^3^ cancer cells per well, either together (coculture) or separately. Six h prior to running the assay, 1.2 × 10^4^ neutrophils per well were added and DMEM without neutrophils was added to wells with coculture and monocultures as controls. The day prior to the assay, sensor cartridges were hydrated with 200 µl of deionized water in the sensor cartridge plate for 12 h in a non-CO_2_ incubator at 37 °C. On the morning of the assay, the deionized water was removed and replaced with 200 µl of calibration buffer. MitoStress assay media (low buffered DMEM; Merck/Sigma Aldrich) was supplemented with glutamine (2 mM), glucose (10 mM) and pyruvate (1mM from a freshly made 1 M stock solution). GlycoStress assay media consisted of DMEM supplemented with glutamine (2 mM). Media were incubated in a non-CO_2_ incubator at 37 °C and adjusted to pH 7.4 using 1 M NaOH. Growth medium was removed, and cells were washed thrice with 200 µl assay media per well, prior to incubation with 180 µl assay media per well in a non-CO_2_ incubator for 60 min at 37 °C prior to the assay. For MitoStress assays, oligomycin (1 µM), carbonyl cyanide 4-(trifluoromethoxy)phenylhydrazone (FCCP; 0.5 µM) and rotenone/antimycin A (0.5 µM each) were used. For GlycoStress assay, glucose (10 mM), oligomycin (1 µM) and 2 deoxy glucose (50 mM) were used. Each measurement cycle consisted of 3 min. Three baseline measurements were taken prior to the addition of each compound, and subsequently, 3 measurements were taken afterwards. Oxygen consumption rate (OCR) and extracellular acidification rate (ECAR) were reported as absolute rates (pmoles minute^− 1^ for OCR and mpH minute^− 1^ for ECAR) or normalized to cells number. Normalization was achieved following completion of the Seahorse assay using Hoechst 33,342 staining. Hoechst 33,342 was diluted in in serum-free media at a concentration of 5 µg ml^− 1^ (10 × working concentration) and 10 µl were dispensed into each well. After 10 min incubation, fluorescence was measured (excitation 405 nm and emission 450 nm) using a plate reader (Synergy 2, BioTek). Numbers used for normalization are presented in Supplementary Table 1. Data were exported from Seahorse Wave software into the Seahorse XF Report Generator (Agilent). Biological and technical replicates for each condition (3 technical replicates for each of the 3 biological replicates) were compiled in GraphPad Prism.

### Flow cytometry

To assess cell survival in monocultures and cocultures, GFP-expressing fibroblasts and mCherry-expressing cancer cells were seeded in 6-well plates at a total density of 3 × 10^5^ cells per well. Monocultures contained 3 × 10^5^ fibroblasts or 3 × 10^5^ cancer cells, whereas cocultures contained 2 × 10^5^ fibroblasts together with 1 × 10^5^ cancer cells. Samples were run with one technical replicate per condition. After 72 h, 3 × 10^5^ neutrophils were added to each well. After 6 h, cells were detached using trypsin/EDTA. Conditioned media and cells were collected in cell culture medium and centrifuged at 300 × g at 4 °C. Each pellet was resuspended in PBS and stained with LIVE/DEAD Fixable Violet Dead Cell Stain (#34963 Thermo Fisher Scientific) according to the manufacturer’s recommendations. Neutrophils were stained with APC-conjugated anti-CD66 antibody (#305117 BioLegend). Samples were evaluated using a ZE5 Cell Analyzer (Bio-Rad). Gating was performed according to forward and side scatter, and APC-positive cells were identified as neutrophils, GFP-positive cells as fibroblasts, and mCherry-positive cells as cancer cells. Data were processed using FlowJo software v10.7.1 (BD Biosciences).

To evaluate cell growth, 3.3 × 10^4^ cancer cells were seeded in 12-well plates together with 6.6 × 10^4^ fibroblasts to establish cocultures. Cancer cells and fibroblasts cultured alone served as controls. Samples were run with one technical replicate per condition. After two days of culture, medium was removed and 1 × 10^5^ neutrophils were added to coculture and corresponding control wells. After 3 days, samples were prepared for flow cytometry. Conditioned media were collected, and cells were removed from the wells using trypsin/EDTA. The contents of each well were transferred to Eppendorf tubes and centrifuged for 5 min at 300 × g. The medium was removed, and each pellet was resuspended in PBS. Flow cytometry was performed using a ZE5 Cell Analyzer (Bio-Rad), and all cells in each sample were counted. Data were evaluated using FlowJo software v10.7.1 (BD Biosciences), and cells were classified according to mCherry expression (cancer cells) and GFP expression (fibroblasts). Results are presented as the percentage increase after neutrophil addition compared with samples without neutrophils.

### DNA fluorescence assay

Cells were seeded in 96-well plates as fibroblast monocultures (8 × 10^3^ cells), cancer cell monocultures (4 × 10^3^ cells), or cocultures containing 8 × 10^3^ fibroblasts together with 4 × 10^3^ cancer cells and incubated for 72 h. After incubation, 5 × 104^4^ neutrophils per well were added together with _2_.5 µM Sytox Green nucleic acid stain (Thermo Fisher Scientific). Cell culture media without neutrophils were added to control wells. Triton solution (1%) was added to wells serving as maximum lysis controls, which were set as 100% for calculations. After 15 min of incubation at 37 °C with 5% CO_2_, 100 nM PMA was added to wells containing neutrophils alone as a positive control. Unstimulated neutrophils served as a negative control. Fluorescence intensity (FI) was measured using a plate reader (FLUOstar OMEGA; BMG Labtech) at 37 °C with 5% CO_2_. Signal intensity (excitation 485 nm, emission 520 nm) was measured every 10 min for 10 h. Results are presented as FI, calculated as the mean of six technical replicates over time and expressed as a percentage of the maximum level derived from lysis controls.

### Measurement of glucose and lactate

Glucose and lactate were measured in conditioned media from monocultures or cocultures in the presence or absence of neutrophils. The conditioned media were collected before neutrophil addition, or after 6 h of incubation. Samples were run with one technical replicate per condition. The measurement was performed by the Clinical Chemistry Laboratory at Umeå University Hospital using a Cobas Pro-c503 analytical unit. For the measurement of glucose, gluc3 (Roche Diagnostics Scandinavia AB) was used as reagent and the concentration of glucose was estimated photometrically. For lactate, LACT2 (Roche diagnostics Scandinavia AB) was used as reagent and lactate was determined according to the increase of absorbance, wavelength 700/660 nm (sub/main).

### Statistics

GraphPad Prism VI was used for statistical analysis. Unless otherwise stated, one-way ANOVA with Tukey’s multiple comparison test was used. The Kruskal–Wallis test was used when data were not normally distributed. P values < 0.05 were considered statistically significant and marked with one star (*), p values < 0.01 with two stars (**), p values < 0.001 with 3 stars (***), and p values <0.0001 with 4 stars (****). The same labeling of statistical significance was used throughout the study.

## Results

### Conditioned media from cocultures show a pro-inflammatory profile

**Fig. 1:**
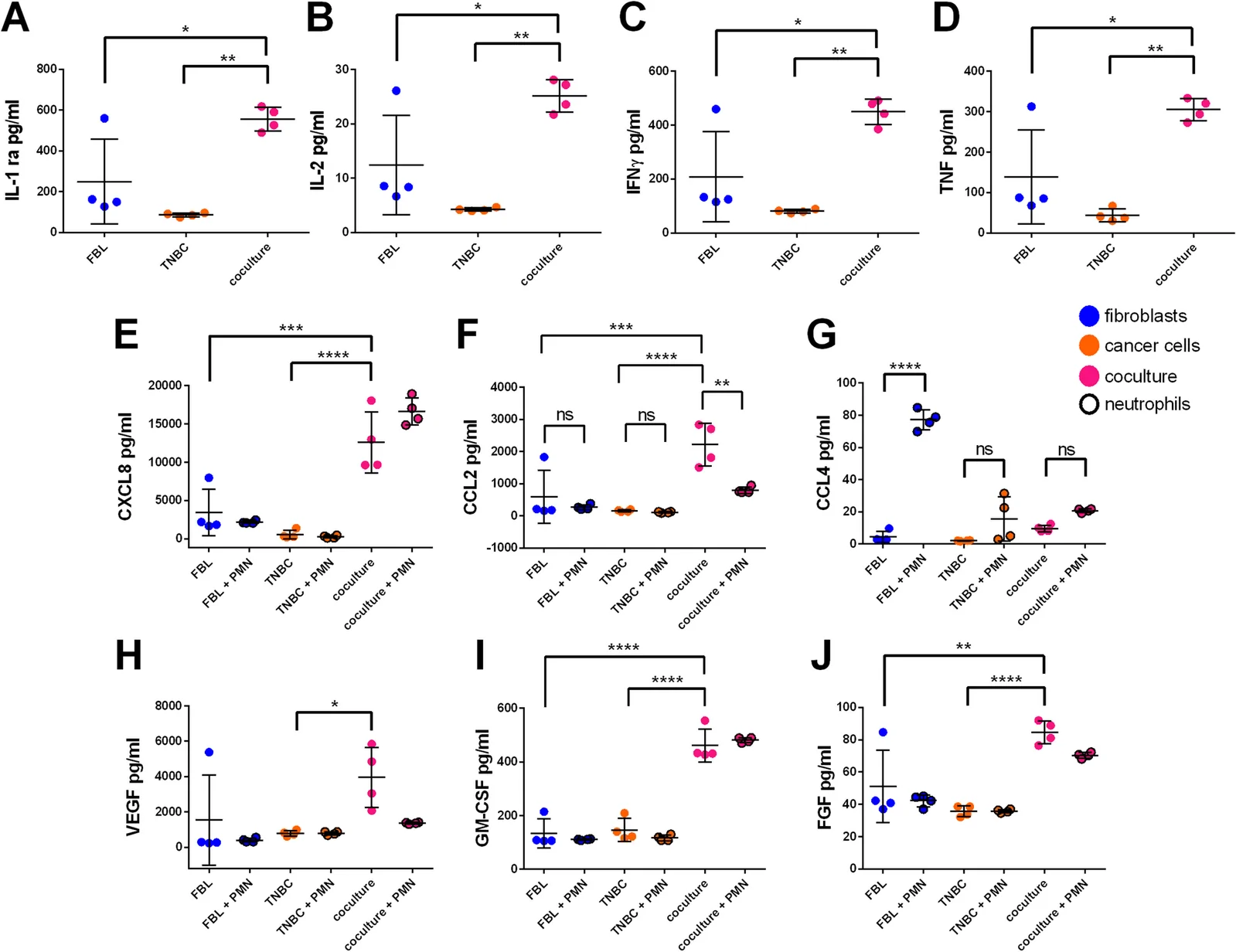
Pro-inflammatory cytokine release by cocultures and modest cytokine changes upon neutrophil addition. Monocultures and cocultures were incubated for three days without neutrophils. The figure shows levels of IL-1ra (**A**), IL-2 (**B**), IFN-γ (**C**), and TNF (**D**). Cytokine levels did not differ significantly between monocultures, whereas cocultures showed significantly elevated cytokine levels. Cytokines were measured in conditioned media collected 6 hours after neutrophil addition (**E**–**J**). Cultures without neutrophils served as controls. Chemokine levels were increased in cocultures compared with monocultures (CXCL8, E; CCL2, F). CCL2 levels decreased in cocultures after neutrophil addition (**F**), whereas CCL4 increased significantly in conditioned media from fibroblasts after neutrophil addition (**C**). Growth factor levels were increased in cocultures but not in monocultures (VEGF, H; GM-CSF,I; FGF,J). FBL = fibroblasts, TNBC = MDA_MB-231 cell line, PMN = neutrophils. Four biological replicates with one technical replicate per condition were used. Statistical analysis was performed using one-way ANOVA. * represents p < 0.05, ** p < 0.01, *** p < 0.001, **** p < 0.0001, and ns = not significant

We measured cytokine release in monocultures and cocultures 6 h after neutrophil addition. Monocultures and cocultures without neutrophils were used as controls. Pro-inflammatory cytokine release was higher in cocultures than in monocultures, in line with previous findings showing that cancer cell–stromal cell interactions can generate a pro-inflammatory environment [29] (Fig. 1).

When evaluating chemokine levels in conditioned media, we observed increased IL-8/CXCL8 secretion from fibroblast monocultures, with even higher CXCL8 levels in cocultures (Fig. 1E). Similarly, MCP-1/CCL2 levels were higher in cocultures than in monocultures (Fig. 1F), but decreased after neutrophil addition (Fig. 1F). In contrast, MIP-1β/CCL4 levels increased in conditioned media from fibroblasts after neutrophil addition, whereas no comparable change was observed in cancer cell monocultures or cocultures (Fig. 1G). Notably, neutrophil monocultures released negligible amounts of the cytokines quantified here (Supplementary Table 1). Growth factor release was also increased in conditioned media from cocultures compared with monocultures (Fig. 1H**–**J). These findings support that fibroblast–cancer cell coculture induces a pro-inflammatory environment. However, neutrophil addition altered the cytokine profile only modestly, indicating that the coculture itself is the main driver of the inflammatory milieu in this model.

### Neutrophils are activated by coculture-conditioned media

**Fig. 2:**
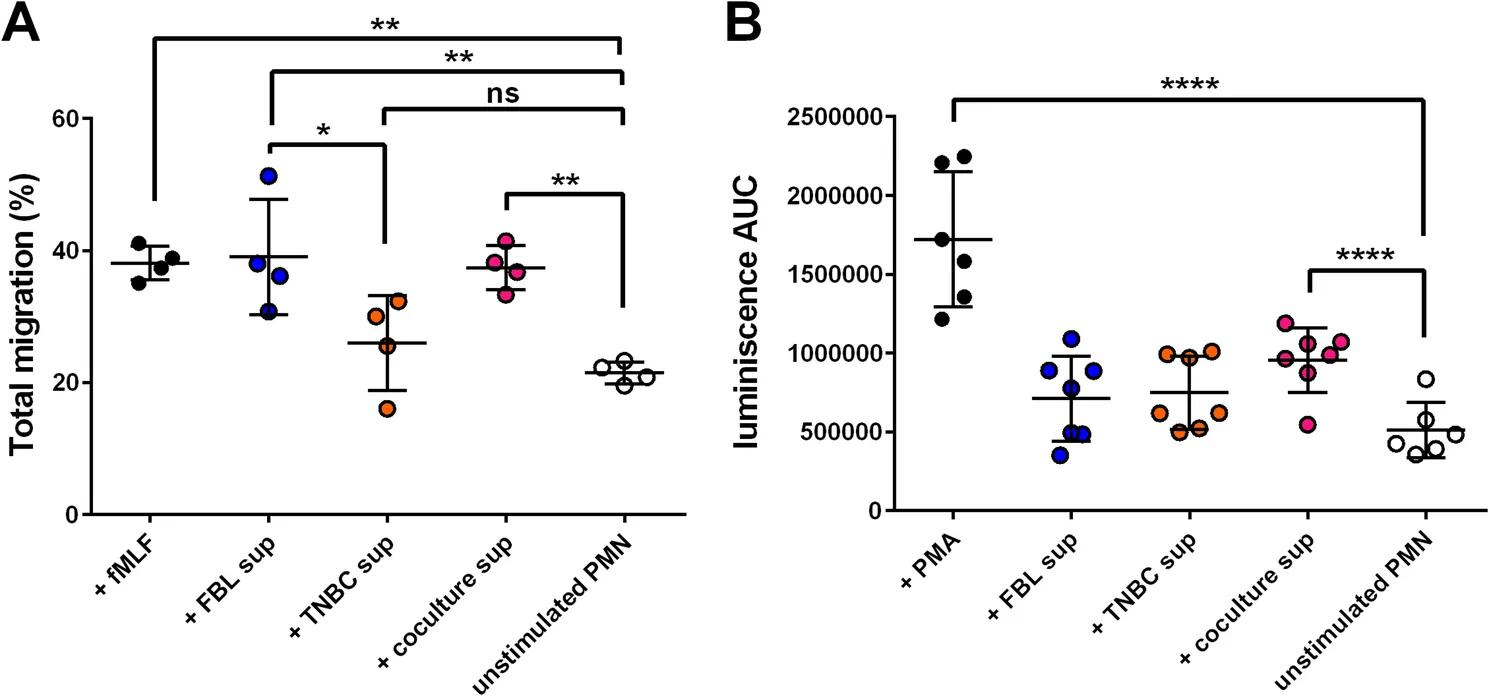
Neutrophils migrate toward and are activated by conditioned media from cocultures. Chemotaxis toward fMLF or conditioned media from cell cultures is shown as total migration, expressed as the percentage of maximum migration after 30 min (**A**). Four biological replicates were used, with two technical replicates for fMLF and one technical replicate for the other conditions. Conditioned media from fibroblast monocultures and cocultures attracted neutrophils to levels similar to fMLF. Chemotaxis toward conditioned media from cancer cell monocultures was not significantly higher than chemotaxis toward medium alone. ROS production is presented as the area under the luminescence curve measured over 3 h (**B**). ROS production increased in neutrophils exposed to conditioned media from cocultures, whereas ROS production in neutrophils exposed to conditioned media from fibroblast or cancer cell monocultures remained similar to the unstimulated control. FBL sup = conditioned media from fibroblasts, TNBC sup = conditioned media from MDA_MB-231 cell line, PMN = neutrophils. Six biological replicates were used for PMA and unstimulated controls, and 7 biological replicates were used for the other conditions, with 5 technical replicates per condition. Statistical analysis was performed using one-way ANOVA. * represents p < 0.05, ** p < 0.01, and **** p < 0.0001

Neutrophil chemotaxis increased toward conditioned media from cocultures and fibroblast monocultures (Fig. 2A). This is likely related to increased CXCL8 levels detected, because CXCL8 is a potent neutrophil chemoattractant (Fig. 1E**–**G). In contrast, conditioned media from cancer cell monocultures did not attract neutrophils more strongly than medium alone.

To better understand interactions among neutrophils, fibroblasts, and cancer cells, we assessed neutrophil activation by quantifying ROS production, a hallmark of neutrophil stimulation. ROS was measured using a luminescence-based assay in neutrophils exposed to conditioned media from monocultures, cocultures, or medium alone as a baseline control. PMA-treated neutrophils served as a positive control. Neutrophils exposed to coculture-conditioned media showed increased ROS production, whereas conditioned media from monocultures induced only non-significant trends compared with baseline levels (Fig. 2B). These findings suggest that neutrophils are recruited and activated by the pro-inflammatory environment generated in cocultures, as shown by increased chemotaxis and ROS production after exposure to coculture-conditioned media.

### Neutrophils do not affect growth or survival of cancer cells in vitro

**Fig. 3:**
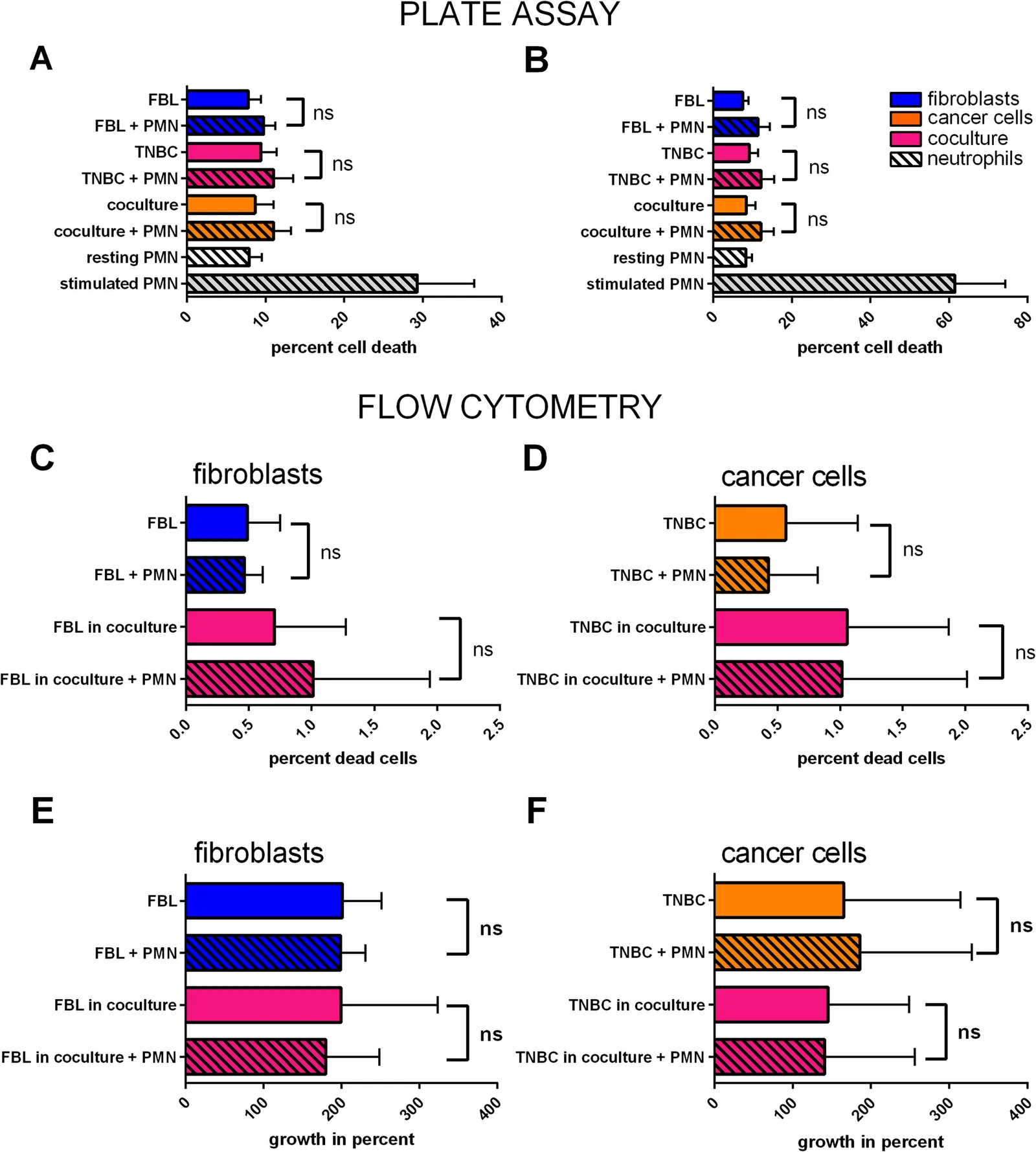
Cell viability and growth are not altered by neutrophil addition. Cell death was quantified using the membrane-impermeable nucleic acid dye Sytox Green in fibroblasts (FBL), cancer cells (TNBC) and cocultures 3 hours (**A**) and 6 hours (**B**) with and without neutrophil (PMN) addition. Phorbol 12-myristate 13-acetate (PMA) stimulation was used for neutrophils alone as a positive control for cell death. No increase in cell death was observed after neutrophil addition. Eight biological replicates with six technical replicates per condition were used. To quantify live and dead cells simultaneously, cells were labeled with a fluorescence-based LIVE/DEAD stain and analyzed by flow cytometry (**C**–**F**). Nonviable fibroblasts (**C**) and cancer cells (**D**) were quantified 6 hours after neutrophil addition and remained unchanged in monocultures and cocultures. Three biological replicates with one technical replicate per condition were used. Viable fibroblasts (**E**) and cancer cells (**F**) were quantified as percentage increase in live cell count relative to baseline. Neutrophil addition did not significantly affect viable cell counts. FBL = fibroblasts, TNBC = MDA_MB-231 cell line, PMN = neutrophils. Three to 4 biological replicates with one technical replicate per condition were used. Statistical analysis was performed using one-way ANOVA and ‘ns’ represents statistically not significant

After determining that neutrophils were attracted to and activated by the coculture system, we assessed whether neutrophils affected cell survival. Cell death was quantified using a fluorescence-based assay (see Methods). Neutrophil addition did not change the proportion of dead cells in monocultures or cocultures after either 3 h (Fig. 3A) or 6 h (Fig. 3B) of incubation. PMA-stimulated neutrophils served as a positive control. Using the aforementioned cell death assay, we were unable to assess any changes in the number of growing cells. Hence, we assessed cell cultures using a LIVE/DEAD staining kit and recorded changes in numbers of viable cells by flow cytometry 6 h after neutrophil addition. Fibroblasts expressed GFP, cancer cells expressed mCherry, and neutrophils were identified using an APC-labelled anti-CD66 antibody. Consistent with the fluorescence-based cell death assay, neutrophil addition did not alter the viability of fibroblasts or cancer cells in neither monoculture nor coculture conditions (Fig. 3C and D).

To evaluate growth rates of the different cell types in cocultures with and without neutrophils, we performed flow cytometry as described in Methods. Neutrophil addition did not alter the growth rate of either cancer cells or fibroblasts (Fig. 3E and F).

Together, these data indicate that although neutrophils mount a measurable response when added to the cultures, they do not affect cell survival or growth under these experimental conditions.

### Neutrophils do not alter the glycolytic metabolic profile but increase the mitochondrial metabolic profile of the coculture system

**Fig. 4:**
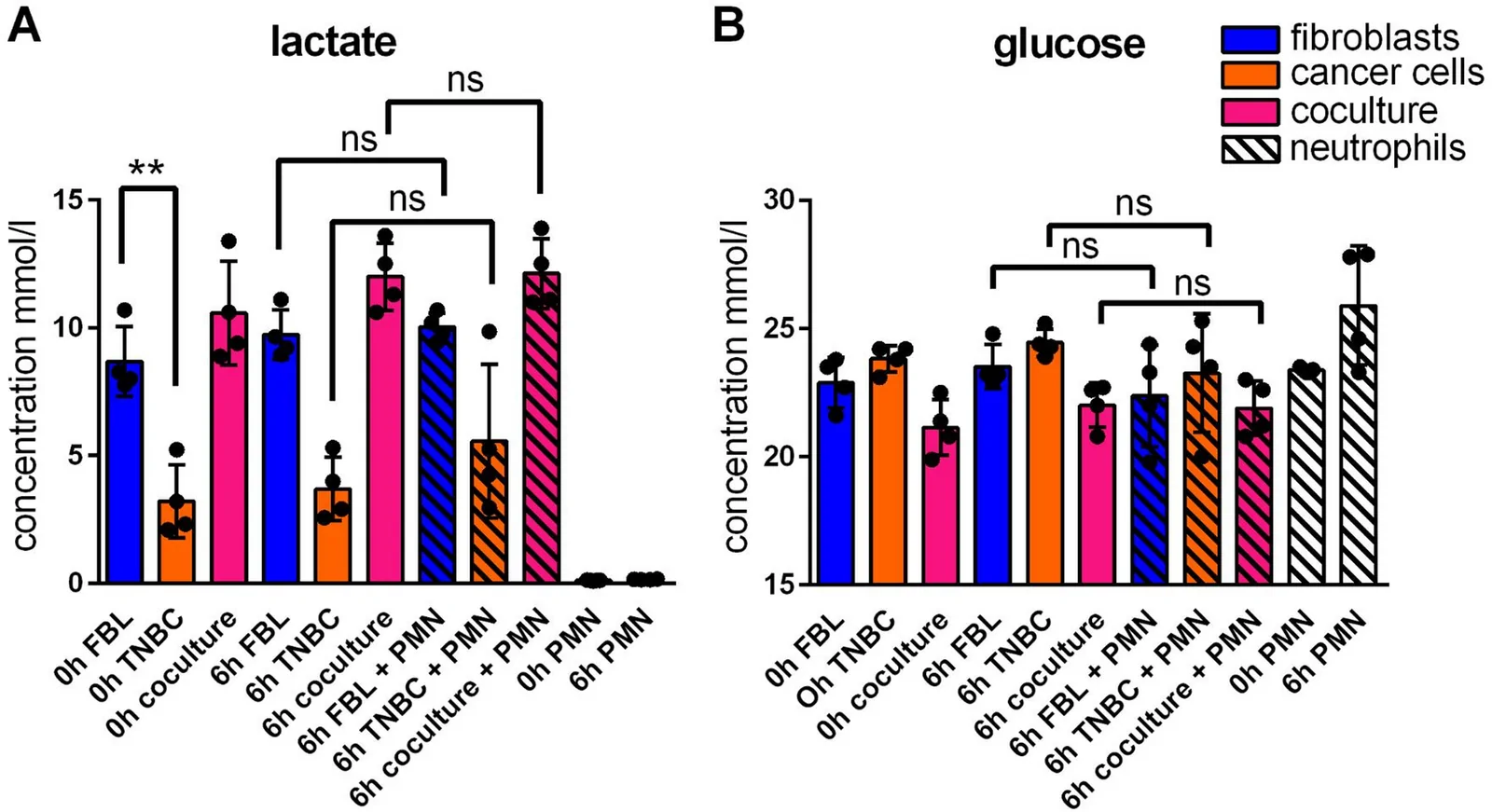
Lactate and glucose levels in conditioned media. Lactate (**A**) and glucose (**B**) were measured in conditioned media collected from monocultures and cocultures before neutrophil (PMN) addition and 6 h after neutrophil addition. Conditioned media from cultures without neutrophils served as controls. Higher lactate release and increased glucose consumption indicate higher glycolytic activity. Lactate production was lower in cancer cell monocultures than in fibroblast monocultures and cocultures (**A**). Glucose consumption differed only modestly between conditions (**B**). Neutrophil addition did not alter lactate or glucose levels, indicating that neutrophils did not measurably affect glycolytic activity in this setting. FBL = fibroblasts, TNBC = MDA_MB-231 cell line, PMN = neutrophils. Four biological replicates with one technical replicate per condition were used. Statistical analysis was performed using one-way ANOVA. ** represents *p* < 0.01 and ‘ns’ stands for statistically not significant

Because neutrophil addition did not directly affect cell survival, we next examined whether neutrophils altered cellular metabolism in monocultures or cocultures. Lactate and glucose levels were measured in conditioned media from monocultures and cocultures before and 6 h after neutrophil addition (Fig. 4A and B). Lactate levels were lower in cancer cell monocultures than in fibroblast monocultures and cocultures, indicating higher glycolytic activity in fibroblasts than in cancer cells under these conditions. Glucose levels were similar across culture conditions, and neutrophil addition did not measurably alter glucose and lactate levels.

**Fig. 5:**
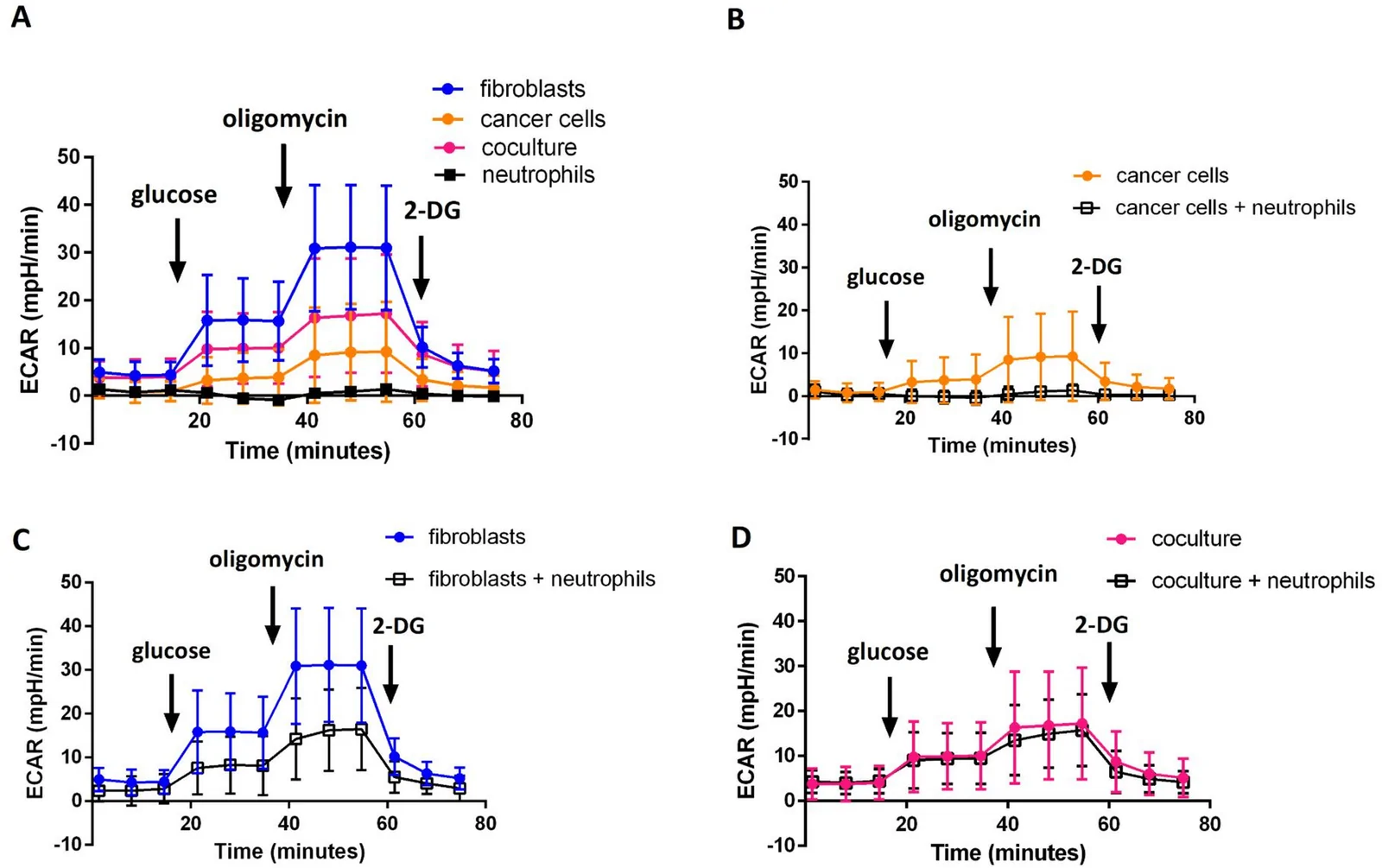
Extracellular acidification rate measured by Seahorse glycolysis stress test. Extracellular acidification rate (ECAR) was quantified using a glycolysis stress test on a Seahorse instrument. Monocultures, cocultures, and neutrophils alone are shown without neutrophil addition (**A**) and after neutrophil addition to monocultures and cocultures (**B**–**D**). Fibroblasts displayed higher glycolytic activity, whereas cancer cells and neutrophils showed lower glycolytic activity. Data were analyzed from 3 biological replicates, representing independent Seahorse runs, with 3 technical replicates for each condition

**Fig. 6:**
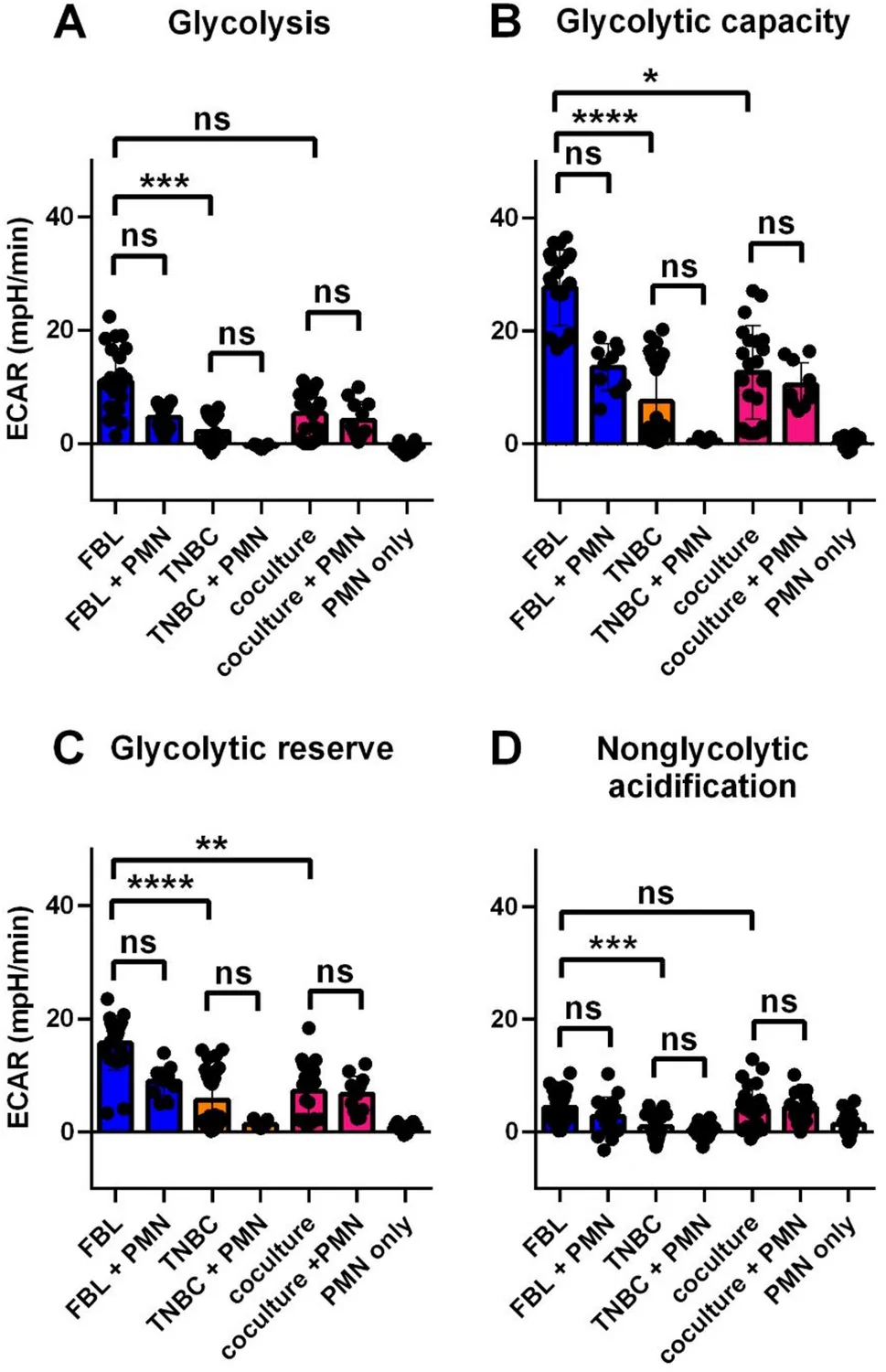
Glycolytic parameters derived from Seahorse analysis. ECAR is presented as glycolysis (**A**), glycolytic capacity (**B**), glycolytic reserve (**C**), and non-glycolytic acidification (**D**). Fibroblasts were more glycolytic than cancer cells (**A**). Glycolytic capacity and glycolytic reserve were decreased in cocultures compared with fibroblast monocultures (**B **and** C**). Fibroblast and cancer cell monocultures showed a non-significant tendency toward reduced glycolytic function after neutrophil addition (**A**–**C**). Non-glycolytic acidification was lower in cancer cells than in fibroblasts. FBL = fibroblasts, TNBC = MDA_MB-231 cell line, PMN = neutrophils. Data were analyzed from 3 biological replicates, representing independent Seahorse runs, with 3 technical replicates for each condition. Statistical analysis was performed using Kruskal–Wallis tests because the data were not normally distributed. * represents *p* < 0.05, ** *p* < 0.01, *** *p* < 0.001, **** *p* < 0.0001, and ‘ns’ statistically not significant

To further address metabolic activity, we used Seahorse analysis to evaluate the bioenergetic profiles of monocultures and cocultures. A glycolysis stress test was used to assess glycolytic function (Fig. 5). Fibroblasts showed higher extracellular acidification rates (ECAR) than cancer cells, indicating greater glycolytic activity (Fig. 5). Glycolytic capacity and glycolytic reserve were decreased in cocultures compared with fibroblast monocultures (Fig. 6B and C). After neutrophil addition, fibroblasts showed non-significant trends toward decreased glycolytic capacity and glycolytic reserve, and cancer cells showed a non-significant trend toward decreased glycolysis (Fig. 6A–C). These trends were not observed in cocultures. Neutrophils alone showed very low ECAR (Fig. 5A).

**Fig. 7:**
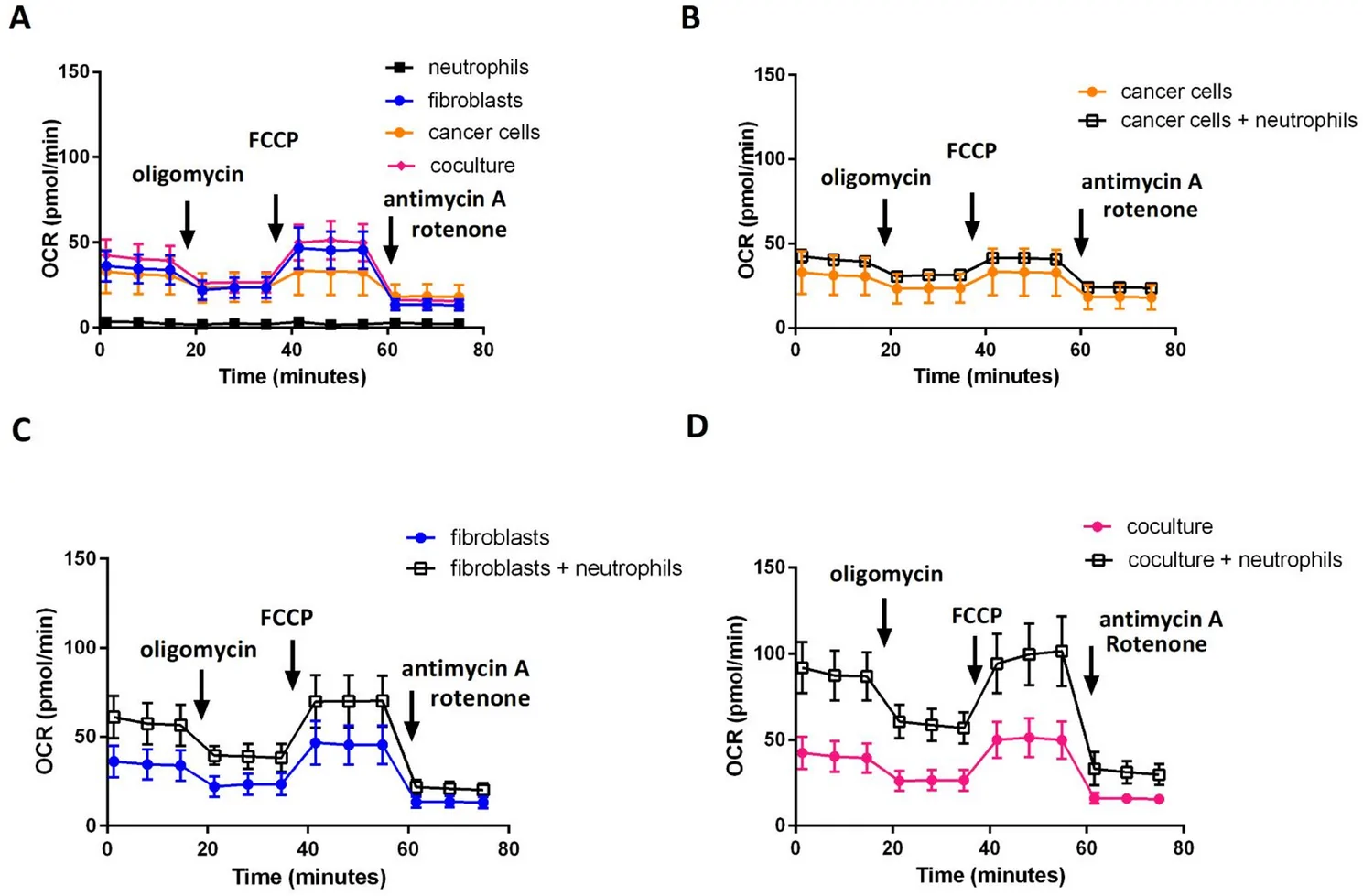
Oxygen consumption rate measured by Seahorse mitochondrial stress test. Oxygen consumption rate (OCR) was measured by Seahorse analysis and is shown for monocultures, cocultures, and neutrophils alone (**A**) and for each culture condition with or without neutrophil addition (**B**–**D**). Mitochondrial metabolism increased in cocultures after neutrophil addition (**D**), an effect not observed in monocultures (**B **and** C**). OCR from neutrophils alone was negligible (**A**). Data were analyzed from 3 biological replicates, representing independent Seahorse runs, with 3 technical replicates for each condition

**Fig. 8:**
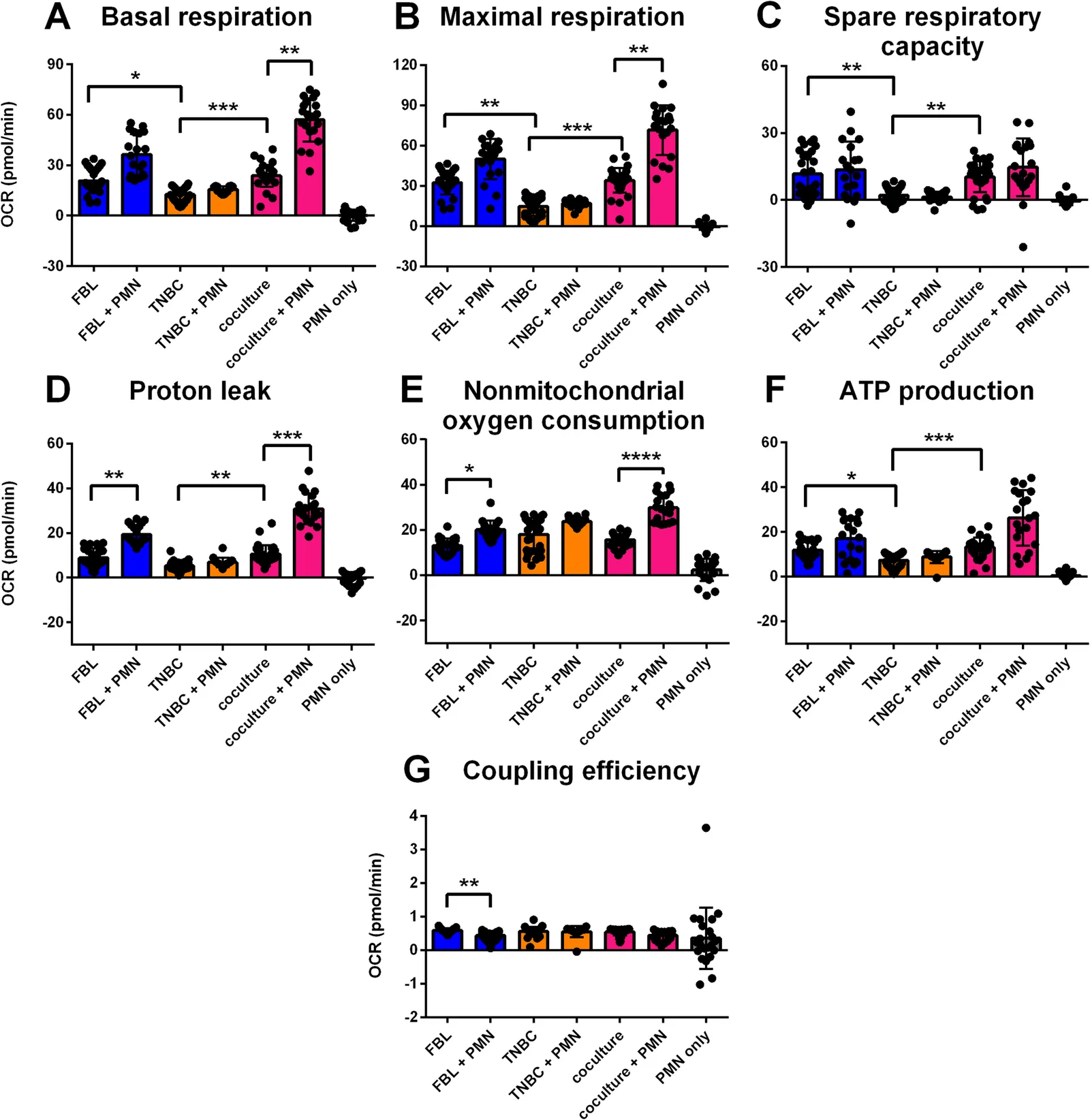
OCR-derived mitochondrial parameters. OCR is presented as basal respiration (**A**), maximal respiration (**B**), spare respiratory capacity (**C**), proton leak (**D**), non-mitochondrial oxygen consumption (**E**), ATP production (**F**), and coupling efficiency (**G**). Basal and maximal respiration increased in cocultures after neutrophil addition. Proton leak and non-mitochondrial oxygen consumption increased in both fibroblast monocultures and cocultures after neutrophil addition. ATP production remained unchanged. Neutrophils reduced coupling efficiency in fibroblasts but did not alter coupling efficiency in cancer cells or cocultures. FBL = fibroblasts, TNBC = MDA_MB-231 cell line, PMN = neutrophils. Data were analyzed from 3 biological replicates, representing independent Seahorse runs, with 3 technical replicates for each condition. Statistical analysis was performed using Kruskal–Wallis tests because the data were not normally distributed. * represents *p* < 0.05, ** *p* < 0.01, *** *p* < 0.001, and **** *p* < 0.0001

Mitochondrial energy metabolism was assessed using the mitochondrial stress test (Fig. 7). Oxygen consumption rate (OCR) in cocultures was similar to that in fibroblast monocultures, and both conditions showed higher basal respiration, maximal respiration, and spare respiratory capacity than cancer cell monocultures (Fig. 8). After neutrophil addition, OCR-related parameters increased in cocultures, including basal respiration, maximal respiration, proton leak, and non-mitochondrial oxygen consumption (Fig. 8A–F). Fibroblast monocultures showed a non-significant tendency toward increased basal and maximal respiration after neutrophil addition (Fig. 8A and B), whereas proton leak and non-mitochondrial oxygen consumption increased significantly (Fig. 8D and F). Neutrophils alone showed negligible mitochondrial activity (Fig. 7A). Overall, these data indicate that neutrophils alter the bioenergetic profile of cocultures by increasing mitochondrial activity, an effect not observed in cancer cell or fibroblast monocultures.

## Discussion

Coculturing a TNBC cell line with fibroblasts generates a pro-inflammatory cytokine milieu (Fig. 1). Here, we used the aggressive and highly metastatic TNBC cell line MDA-MB-231 derived from a pleural effusion. These cells have been extensively characterized and, as a consequence, have been classified as a basal-like TNBC cell line [22]. For broader implications additional cancer cell lines should be used, however our study serves as a good starting point for elucidating metabolic effects of neutrophils in TNBC. Nevertheless, our findings confirm previous reports showing that interactions of cancer and stromal cells generate a pro-inflammatory environment [29] supporting the notion that inflammation contributes to tumorigenesis [30]. Patients with advanced cancer often present with neutrophilia and elevated neutrophil-to-lymphocyte ratios, both of which are known negative prognostic factors [31]. In addition, high circulating levels of IL-6, CXCL8, IL-10, CCL2, and IFN-γ in patients with metastatic breast cancer are linked to worse prognosis [32]. We found elevated levels of IFN-γ, CXCL8, and CCL2 in conditioned media from cocultures, but not from cancer cells alone (Fig. 1C and Fig. 1E and F). Adding neutrophils to cancer cell monocultures affected cytokine release modestly. In cocultures, neutrophil addition decreased CCL2 levels, whereas CCL4 increased significantly after neutrophil addition to fibroblast cultures (Fig. 1F and G). A similar, non-significant trend of increased CCL4 was observed in cocultures with neutrophils. Notably, CCL4 and CCL2 show redundancy which suggests compensatory effects. Möckel et al. showed that anti-CCL2 treatment in mice with TNBC altered tumor blood vessels, decreased blood volume, and improved delivery of anti-tumor drugs, indicating an anti-cancer effect [33]. This is notable because VEGF levels in our analysis tended to decrease after neutrophil addition to cocultures (Fig. 3D), although not statistically significant.

The chemokine CCL4 has been detected at elevated levels in the stromal compartment of breast cancer tissue and has been suggested to be produced by macrophages in the TME [34]. We observed increased CCL4 levels in conditioned media from fibroblasts exposed to neutrophils (Fig. 2G). However, the present experimental design does not allow us to determine which cell type produced the chemokine. Cathepsin D, a protease that degrades CCL4, is produced by MCF7 cells, MDA-MB-231 cells, and TNBC tissue [34, 35]. Because we did not measure cathepsin D levels, its potential role in the lower CCL4 levels observed in cancer cell cultures and cocultures exposed to neutrophils remains uncertain. The cytokine analysis also showed elevated growth factor levels in conditioned media from cocultures compared with monocultures (Fig. 1H**–J**). Overall, the cytokine analysis suggests that the coculture system reflects selected aspects of the in vivo TME. The neutrophils used in this study were isolated from healthy donors using a method that yields highly pure mature normal-density neutrophils [28]. Hence, they are unlikely to fully represent neutrophil phenotypes present in patients with ongoing cancer. However, our model may reflect aspects of early tumor development before a disease-associated neutrophil phenotype emerges.

The increased chemokine levels in conditioned media from cocultures and, to some extent, fibroblast monocultures (Fig. 1E and F) prompted us to test whether these environments were chemoattractant to neutrophils. Neutrophils are present in the breast cancer TME, particularly in TNBC, which has the highest occurrence of tumor-associated neutrophils among breast cancer subtypes [16]. Chemotaxis increased toward conditioned media from cocultures and fibroblast monocultures, but not toward conditioned media from cancer cells alone (Fig. 2A).

In the cytokine analysis, we found increased CXCL8 levels in conditioned media from both cocultures and fibroblast monocultures. CXCL8 is a potent neutrophil chemoattractant, and its presence in conditioned media from fibroblasts may explain the increased neutrophil chemotaxis [36]. These findings suggest that the coculture environment promotes neutrophil recruitment more strongly than cancer cells alone.

The reverse Warburg effect describes how cancer cells can induce stress in surrounding stromal cells, for example through ROS, thereby altering metabolic interactions in ways that favor cancer cells [21]. Because the overall goal of this study was to explore interactions among cancer cells, fibroblasts, and neutrophils, we investigated neutrophil activation under different culture conditions. Using a luminescence-based assay, we evaluated neutrophil ROS production exposed to conditioned media from different mono- and cocultures. Neutrophil ROS production was significantly higher after exposure to coculture-conditioned media (Fig. 2B) compared to unstimulated neutrophils. This indicates that neutrophil activation is more prominent in cocultures than in monocultures.

As neutrophils were activated by the coculture milieu, we evaluated whether neutrophil activation affected cell survival. Neutrophil addition did, however, not affect cell survival under the culture conditions tested here (Fig. 3A–D), suggesting that potential anticancer effects of neutrophils are too subtle or require additional factors lacking in this model. The absence of antibodies could be such a factor limiting antibody-dependent neutrophil cytotoxicity. Similarly, we did not observe increased cell proliferation after neutrophil addition to any culture condition (Fig. 3E and F).

To identify more subtle changes in metabolic pathways of cancer and stromal cells upon neutrophil addition, we assessed cellular metabolism under the different culture conditions. Seahorse assays confirmed that the bioenergetic profiles of the cell types differed and that coculture further altered metabolic activity, with increased mitochondrial function. Cancer cells showed lower mitochondrial and glycolytic activity than fibroblasts (Figs. 5A, 6A–C and 7A, and Fig. 8A–F), consistent with lactate measurements in conditioned media (Fig. 4A). Cocultures showed mitochondrial activity similar to fibroblast monocultures, which we interpret as increased mitochondrial activity relative to the expected additive contribution of fibroblasts and cancer cells. In contrast, glycolytic function decreased in cocultures, which may reflect the lower glycolytic activity of cancer cells compared with fibroblasts. As expected, non-dividing neutrophils alone showed negligible metabolic activity (Fig. 5A and Fig. 7A). After neutrophil addition, fibroblast and cancer cell monocultures showed non-significant tendencies toward decreased glycolytic activity, whereas this trend was not observed in cocultures. This may indicate that cocultures buffer or compensate for potential neutrophil-driven effects on glycolysis. In contrast, mitochondrial activity increased significantly in cocultures after neutrophil addition, an effect absent in monocultures. Fibroblasts can respond to chemotherapy exposure with increased glycolysis, which may support tumor metastasis [37]. MDA-MB-231 cells rely on both mitochondrial activity and glycolysis [38], consistent with the metabolic pattern observed here. Macrophages can induce aerobic glycolysis in fibroblasts through TNF production [39], indicating that a pro-inflammatory environment can affect stromal cell metabolism. Since neutrophils release more ROS upon exposure to cocultures but not increased levels of TNF, ROS may contribute to the neutrophil-associated metabolic changes observed in our study.

## Conclusions

Coculturing MDA-MB-231 cells with stromal fibroblasts generates a more physiological microenvironment than cancer cell monocultures. Neutrophils from healthy donors were attracted to and activated by this coculture environment, but they did not directly affect cell survival or growth. Coculturing altered the metabolic profiles of the cells, and neutrophils additionally increased mitochondrial activity in the coculture system. This model may therefore reflect some aspects of early tumor–neutrophil interactions, while not capturing neutrophil phenotypes associated with later-stage tumor progression. Together, our findings suggest that neutrophils can influence the metabolic state of MDA-MB-231 cells in cocultures and provide a basis for further studies on neutrophil–cancer cell interactions in more complex and clinically relevant models.

## Supplementary information

Below is the link to the electronic supplementary material.

Supplementary Material 1

## Data Availability

The datasets used and/or analysed during the current study are available from the corresponding author on reasonable request.
