# Supplementary material for: Neutrophils alter the metabolic profile of a coculture model of triple-negative breast cancer and stromal cells
Source: BMC Cancer. 2026 Oct 22;26:1272. doi: 10.1186/s12885-026-17186-6 (PMC13693121; doi:10.1186/s12885-026-17186-6)
Supplement: Supplementary file 1 [file 12885_2026_17186_MOESM1_ESM.pdf]

Supplementary tables Salzer *et al*., 2026

Average cell numbers used for Seahorse experiments.

Fibroblast average cell number	5525
Cancer cells average cell number	4933
Coculture average cell number	7020
Neutrophil average cell number	1724
Fibroblasts + neutrophils average cell number	3294
Cancer cells + neutrophils average cell number	5077
Coculture + neutrophils	3586

Supplementary table 1: Values used for normalization of Seahorse data. Data were generated using Hoechst 33342 staining performed after completion of the Seahorse assay.

Cytokine levels in conditioned media from unstimulated neutrophils.

Cytokine	pg/ml	pg/ml	pg/ml
IL-1 ra	7.95	*	*
IL-2	*	*	*
IFN-γ	*	*	*
TNF	*	*	*
CXCL8	5.01	4.03	4.03
CCL2	*	*	*
CCL4	0.49	*	*
VEGF	5.31	7,89	6,17
GM-CSF	8.40	9.23	8.37
FGF	16.61	15.26	19.13

Supplementary table 2: Cytokines measured in supernatant collected after neutrophils alone were added to wells in absence of cancer cells or fibroblasts. The symbol * represents levels below detection limit of the respective cytokine. Notably, all levels were below levels quantified in supernatants collected from cancer cells and fibroblasts and consequently considered negligible. Three biological replicates with one technical replicate per condition were used.
